# The Role of Self-esteem, Self-efficacy, Social Support and Resilience in Psychological Adjustment in Schoolchildren

**DOI:** 10.11621/pir.2025.0101

**Published:** 2025-03-01

**Authors:** Norma Ivonne González-Arratia López-Fuentes, Martha Adelina Torres Muñoz, Rolando Díaz-Loving

**Affiliations:** a Autonomous Mexico State University, Mexico; b National Autonomous University of Mexico, Mexico

**Keywords:** psychological resilience, psychological adjustment, childhood, vulnerability, protective factors

## Abstract

**Background:**

There has been a recent increase in research to empirically verify different personal and contextual variables that impact psychological adjustment indicators, but further research is still needed in the construction of explanatory models, especially for children.

**Objective:**

This study aimed to analyze the role of self-esteem, self-efficacy, and family social support in resilience, and their effect on indicators of psychological adjustment, in children living in at-risk contexts.

**Design:**

A sample of 450 participants (229 boys, 221 girls) aged 9 to 12 years, with a mean age of 1.70 (SD = .67), participated in the study. With the parents’ consent, the children completed a questionnaire containing sociodemographic questions and seven scales for the measurement of each of the variables under study.

**Results:**

Significant differences in the predictor variables were found according to the levels of resilience, but not with respect to gender, and the correlations between the variables were found to be significant. The proposed structural model was verified, which shows acceptable fit indices and highlights that family social support is related to resilience and psychological adjustment.

**Conclusion:**

Personality characteristics and family social support, as personal and social variables, constitute protective factors during childhood in the context of psychosocial risk, suggesting that they must be taken into account when implementing programs to promote resilience and well-being.

## Introduction

Resilience is an important topic in positive psychology (Gínez-Silva et al., 2019) that has been conceptualized in multiple ways. Most researchers define it as a set of personal qualities that lead to spiritual growth and development in the face of adversity, resulting in optimal functioning after having overcome one or more traumatic events (Morán-Astorga et al., 2019). Other authors define resilience as a result of a dynamic and evolutionary process that may vary according to individual circumstances, the nature of the trauma, and the context and stage of life (Cyrulnik, 2001). In Bronfenbrenner’s (1979) ecological model, emphasis is placed on the relationships that individuals have with their environment, allowing for successful adaptation. Thus, based on this model, resilience is defined in this study as the ability to face and recover from stressful situations and demands of the environment; it is dynamic in the sense that it implies an interaction between the processes of risk and protection, both internal and external to the individual, which are put into play to modify the effects of adverse events (González-Arratia et al., 2022).

Throughout life, people are presented with at least one event that can be considered potentially traumatic (Bonanno & Mancinni, 2008), so different psychological resources must be mobilized to facilitate adaptation to the environment and, thus, ensure psychological adjustment (Cobos-Sánchez et al., 2016). In the case of children and adolescents, there is greater vulnerability due to inadequate psychological development that arises from the risk conditions associated with physical and emotional changes, as well as social and contextual changes such as exposure to violence, neglect, poverty, family dysfunction, and neglectful parenting style or overly demanding parents who can negatively affect their child’s behavior (Martínez-Cárdenas & González-Sábado, 2017). In addition, according to Masten and Reed (2002), with the accumulation of biological, cognitive, and environmental changes, along with their interaction, new conflicts that arise during this development period result in a more vulnerable situation (Steingberg et al., 2006). Adolescents living in contexts of social vulnerability such as poverty may find it difficult to overcome to adversity if they have low levels of psychosocial adjustment. However, “they can develop resources that allow them to cope with adverse conditions” (Díaz & Morales, 2021, p. 3).

It has been observed that despite living in situations of adversity and/or risk, people can cope with and even overcome such adversity if they have protective factors, which can be individual factors, such as psychosocial adjustment, familial factors, or social factors, that cushion the impact of psychosocial risk. This is usually understood as being equivalent to adaptation and “indicates the appropriate response to the different situations and demands of the surrounding context” (Madariaga et al., 2014, p. 305).

Sanmarco et al. (2019) define resilience as the ability to use coping strategies that are aimed at maintaining an optimal level of functioning and a balance between internal and external needs; therefore, this definition implies that there is an efficient use of available material and psychological resources. In addition, psychological adjustment is linked to the surrounding context, as well as to some personality characteristics such as self-esteem, self-efficacy, and family social support (Gutiérrez & Pastor, 2021).

To achieve psychological adjustment, there must be a balance in the emotional, cognitive, and social aspects of the individual, which, consequently, leads to well-being; however, if there is psychological maladjustment, the probability of experiencing discomfort and behavioral problems increases (Cobos-Sánchez et al., 2016; Gavazzi, 2013; Fuentes et al., 2015). The model proposed by Madariaga et al. (2014) indicates that psychosocial adjustment is facilitated by the presence of “psychological variables such as self-concept, emotional intelligence, social skills that have their expression in satisfaction or well-being and entail the absence of antisocial behaviors or psychological symptoms of a clinical nature” (p. 305).

Among the psychological variables associated with resilience, self-esteem stands out as a determinant of psychological adjustment (Gómez-Baya et al., 2019; Gökmen, 2016). Froxán et al. (2020) define self-esteem as the set of verbalizations and assessments with which a person describes themselves. These verbalizations are part of the judgment towards oneself and are made up of adaptive or dysfunctional thoughts that elicit behaviors through which positive or negative emotions are experienced. High self-esteem allows an individual to face stressful situations when living in poverty with a better attitude (González-Arratia, 2018) and successfully overcome difficulties; it predicts psychosocial adjustment as it is related to fewer emotional and behavioral problems (Schoeps et al., 2019; Rolandi, 2023). In the same way, self-esteem allows individuals to effectively manage stressful situations (Mur et al., 2023), predicts resilience (González-Arratia et al., 2022) and facilitates psychosocial adjustment in adolescents facing social vulnerability (Díaz & Morales, 2021).

One of the determinants of psychological adjustment is self-efficacy, which, according to Bandura (2001), is defined as the personal belief in one’s own capabilities when dealing with specific tasks in different situations, which gives people the ability to organize and execute the coping actions necessary to achieve psychological adjustment.

Family social support is an interactive process that people experience with their family members, and it is contingent on how people perceive or experience being loved and valued; it has been reported that support from one’s family and school is a source of satisfaction. Family social support is related to psychological adjustment and emotional well-being among children and adolescents (Gutiérrez & Pastor, 2021). It is a relevant contextual factor that affects the capacity for resilience and psychosocial adjustment, since it constitutes a protective factor against the demands of the environment (Leiva et al., 2013, Rodríguez-Fernández et al., 2015).

It has been reported that resilience and psychological adjustment have a positive relationship with each other; as resilience increases, well-being and psychological adjustment also increase (Ramos-Díaz, 2015; Cerezo & Rueda, 2020; Moreno-López et al., 2019). In terms of the differences between men and women, research has indicated that, during childhood and adolescence, men display greater behavioral problems compared to women, who are more likely to show emotional symptoms; it has also been reported that the older an individual, the better their psychological adjustment (Kökönyei et al., 2015; Ansary et al., 2017).

During the last decade, resilience has been investigated with respect to indicators such as psychological adjustment, life satisfaction (Li et al., 2012), and the experience of positive emotions (Ong et al, 2006). Most of the research has been carried out in Europe and the United States and has been particularly focused on samples of people over 18 years of age and university students. Given the importance of sociocultural variables as determinants of individual patterns (Diaz-Loving, 2019), it is crucial to conduct research in Mexico where there is still little empirical evidence derived from studies that simultaneously include variables such as self-efficacy (Meneghel et al., 2021), subjective well-being (Gutiérrez & Romero, 2014), self-esteem, and social support, which will allow a better understanding of the interactions between them. In this context, the need to study these variables together is due to the multicausal nature of psychological adjustment, which has been previously documented by Gutiérrez and Romero (2014) and Ramos Ramos-Díaz (2015). Above all, it requires further research because psychological adjustment difficulties are among the main problems in family, educational, and health systems (Fonseca-Pedrero et al., 2023), especially during the transition from childhood to adolescence in contexts of poverty (Rodríguez & Uriol, 2023).

Based on the above, a model of resilience is proposed to explain psychological adjustment in childhood in conditions of risk such as economic precariousness. This model includes self-esteem, self-efficacy, and family social support, the relationships among which have been investigated separately in previous studies. Empirical evidence is needed to derive a joint explanation of the interactions between these variables in the case of Mexican children, allowing us to outline both the direction and the degree of contribution of each of the variables on indicators of psychological adjustment. Thus, the objectives are of this study are as follows: 1) to analyze possible differences in the levels of resilience based on the variables evaluated and psychological adjustment; 2) to describe the differences between boys and girls; 3) to analyze the relationships among the variables under study; and 4) to examine the role of self-efficacy, self-esteem, and family social support as determinants of resilience and their effect on various indicators of psychological adjustment, namely satisfaction with life, positive affect, and negative affect, using a structural equation model.

In accordance with the study objectives, the following research questions are addressed: Do the variables evaluated in this study differ according to the levels of resilience reported by the participants? Are there differences between boys and girls? Is there a relationship between the variables under study? Are self-esteem, self-efficacy, and family social support related to resilience and indicators of psychological adjustment? Although the literature includes the absence of behavioral problems as an indicator of adaptation, this study prefers to address positive adjustment factors, which facilitate positive development during childhood and adolescence.

## Methods

### Participants

This study was a cross-sectional study that was correlational in scope. Non-probabilistic convenience sampling was used to recruit 450 basic education students from different public institutions, including 229 (5.9%) boys and 221 girls (49.1%), who were considered to be at psychosocial risk due to their socially vulnerable contexts; these contexts can be understood as a condition that “alludes to a certain fragility in the potential of some social groups as a result of the influence of risk factors” (Morelato et al., 2019, p.207) and are based on the indicators set in the General Law of Social Development (or LGDS in Spanish) by the Consejo Nacional de Evaluación de la Política de Desarrollo Social (CONEVAL, 2022) in Mexico, an institution that establishes guidelines and criteria for the identification and measurement of poverty, considering per capita income, educational lag, access to health services, access to social security, quality and housing spaces, access to basic housing services, access to food, and degree of social cohesion. The participants in this study were living in poverty, defined as when a person does not have sufficient income to meet their needs and exhibits one or more social deficiencies (Rodríguez & Uriol, 2023).

The participants ranged in age from 9 to 12 years (*M* = 1.70, *SD* = .67). Considering the small effect size, a preliminary analysis (*t* = 1.64, *1-β* = 1.00) with 95 confidence indicated that the sample size was sufficient to carry out subsequent analyses. Regarding sociodemographic data, 72.9 of the participants reported living with both parents, followed by 22.7 living with only the mother, 2.4 living with only the father, and a small number of participants reporting that they lived with relatives other than their parents (2.0%). The inclusion criteria were being a student in the fourth to the sixth grade at a basic education institution and having the informed consent of the parents. Participants were excluded if they did not wish to participate or did not complete all the instruments.

### Procedure

#### Questionnaires

1. A sociodemographic data card that asked for information on age, sex, education, and people with whom the participants lived.
2. *A resilience scale* (González-Arratia, 2016) with 32 items and response options ranging from 1 (never) to 5 (always) was used in this study. The scale has three dimensions: internal protective factors, external protective factors, and empathy. It was previously reported to explain 43.3 of the total variance and have a high reliability (α = .91). In this study, the total explained variance was 46.31 and the Cronbach’s alpha coefficient was .93.
3. Subjective well-being assessed with two scales. For the cognitive component, a version of the *Life Satisfaction* (SWLS) scale (Diener et al., 1985) developed by Atienza et al. (2000) was used, with five items and 7 response options; this scale was reported to explain 58.6 of the total variance and have a Cronbach’s alpha coefficient of .87. For the affective component, the *PANAS scale* (Watson et al., 1988) was used; this scale measures positive affect (PA) and negative affect (AN), with 10 items for each dimension and 4 response options (0 = very slightly or not at all to 4 = extremely). It was reported to have a coefficient of reliability of .86 for AP and .84 for AN (Watson et al., 1988). In the present study, Cronbach’s alpha coefficient was .83 for the SWLS scale, .81 for the PA subscale, and .86 for the NA subscale.
4. *The General Self-Efficacy Scale* (Baessler & Schwarzer, 1996) measures a stable feeling of personal competence in being able to effectively handle a variety of situations. It is a unifactorial scale composed of 10 items and 4 response options, and the authors reported a Cronbach’s alpha coefficient of .87 for internal consistency. The Cronbach’s alpha coefficient was .85 for this study.
5. *The Perceived Family Social Support Scale* (González-Ramírez & Landero-Hernández, 2014). The family subscale was applied, consisting of 7 items with 5 response options (1 = never, 5 = always); it was reported to have a Cronbach’s alpha coefficient of .92 and an explained variance of 66.09%. A Cronbach’s alpha coefficient of .86 was obtained for this study.
6. *A self-esteem scale* (González-Arratia, 2011) with 25 items and response options ranging from 4 (Always) to 1 (Never) was used in this study. It has four dimensions: self, family, intellectual work, and affective–emotional, with a reported explained variance of 55.75%. In this study, a Cronbach’s alpha coefficient of .90 was obtained.

With prior authorization from the authorities of each institution, informed consent from the parents and/or guardians, and assent from the participants, various educational centers were visited to administer the study instruments in the respective classrooms according to the academic schedules. The questionnaires were administered through a Google form set up by the researchers, who explained the objectives of the study and addressed any questions or concerns; the duration to answer the questionnaires was one hour.

Descriptive statistics such as mean and standard deviation were analyzed. Univariate normality was checked using Mardia’s (1970) K-S multivariate test for each of the variables, obtaining a significance level of *p*<.001 for all scales, thus showing that none of the variables had a normal distribution. Cronbach’s alpha reliability was analyzed for each scale. A comparison of the levels of resilience was conducted using the Kruskal–Wallis test, and the Mann–Whitney U test was used to analyze differences according to gender. To determine the relationships among the variables, Spearman’s Rho was used. The model was tested using the maximum likelihood method, and the fit of the model was considered using absolute and comparative goodness-of-fit indices according to the criteria of Abad et al. (2011) and Hu and Bentler (1998). According to the recommendations of Abad et al. (2011), different fit indices were considered to evaluate the model; as χ2 is sensitive to sample size, the following indices were therefore included: GFI, AGFI, NFI, CFI, and PGFI with values greater than .90, and the RMSEA index with a value less than .08. The analyses were performed with the IBM-SPSS and AMOS version 25 programs.

## Results

To examine the first study objective, the levels of resilience were determined based on the total scores on this scale; cut-offpoints were determined considering the mean ± 1SD, in which four levels were obtained: group (1)’s scores ranged from 31 to 106 points, indicating very low resilience; group (2)’s scores ranged from 107 to 121 points, indicating moderate resilience; group (3)’s scores ranged from 122 to 135, indicating high resilience; and group (4) with scores higher than 136 could be interpreted as showing very high resilience. Based on the levels of resilience, an analysis of differences was carried out with the Kruskal–Wallis test. The post hoc analysis, using the Games–Howell test, indicated statistically significant differences between groups (1) and (4). In terms of family social support, participants in group 4 had higher scores than those in group 1 (p < .001; 95 CI [–9.03, –4.52]). Statistically significant differences were also observed for self-esteem (95 CI [–21.06, –11.79]) and self-efficacy (95 CI [–12.35, –9.18]). Participants in group 1 with very low resilience had lower PA scores compared to group 4 with very high resilience (95% CI [15.48, –9.06]), but the AN scores were higher for group 1 compared to group 4 (95% CI [2.50, 11.08]). Regarding satisfaction with life, group 4 obtained higher scores (95% CI [–1.76, –5.67]).

*Table 1* shows that group 4 with a higher level of resilience had higher median scores on all variables, except for NA, indicating that they had higher self-esteem and self-efficacy, perceived greater family social support, and showed better psychosocial adjustment. The effect size was calculated based on Cohen’s *d*, indicating a moderate effect for the NA variable and a large effect for the other variables.

**Table 1:** Analysis of differences in the evaluated variables according to the levels of resilience.

	G1 Very low, n = 67	G2 Moderate, n = 118	G3 High, n = 203	G4 Very high, n = 62			
	Mdn (Range)	Mdn (Range)	Mdn (Range)	Mdn (Range)	H	*P*	*Cohen´s d*
Social Support	31(26)	36(35)	40(31)	44(36)	11.49	.001	1.37
Self-esteem	73(49)	83.5(60)	87(35)	89(32)	11.49	.001	1.61
Self-efficacy	28(17)	32(27)	35(21)	40(15)	186.57	.001	3.10
PA	31(26)	36(35)	40(31)	44(36)	111.70	.001	1.75
NA	26(34)	2.5(38)	20(40)	17(40)	33.46	.001	.73
SWLS	26(29)	31(29)	32(30)	35(29)	102.58	.001	1.47

*Note: PA (positive affect), NA (negative affect), and SWLS (satisfaction with life); df = 3, p < .001.*

Regarding the second study objective, an analysis of the differences between boys and girls was carried out for each of the variables using the Mann–Whitney U test. The mean ranges were slightly different between the two groups; however, the differences were not statistically significant, as shown in *Table 2*.

**Table 2:** Analysis of differences between boys and girls for each variable.

	Boys n = 229	Girls n = 221		
	Range	Range	U of Mann–Whitney	*p*
Social Support	236.21	214.40	22,851	.060
Self-esteem	23.32	22.51	24,201	.423
Self-efficacy	228.48	222.41	24,622	.620
PA	229.60	221.25	24,366	.496
NA	226.61	224.35	25,050	.853
SWLS	227.37	223.56	24,876	.754

*Note: PA (positive affect), NA (negative affect), and SWLS (satisfaction with life).*

Prior to testing the model corresponding to the third study objective, a Spearman’s Rho correlation analysis was carried out, which indicated significant low-to-moderate positive or negative relationships for all variables, except for the relationship between PA and NA. *Table 3* shows that the correlation between resilience and self-efficacy is high (Rho = .67, *p* = .001), while the lowest correlation obtained is between resilience and negative affect (Rho = –.28, *p* = .001).

**Table 3:** Intercorrelations, averages, and standard deviations for the total scores of the evaluated variables.

	1	2	3	4	5	6	7	*M*	*SD*	*α*
1 Resilience	1							121.28	14.43	.931
2 Social Support	.52**	1						31.93	4.58	.865
3 Self-esteem	.50**	.57**	1					82.33	9.94	.903
4 Self-efficacy	.67**	.41**	.39**	1				33.17	5.01	.852
5 PA	.55**	.40**	.44**	.49**	1			37.37	7.52	.815
6 NA	–.28**	–.29**	–.34	–.21**	–.04	1		22.88	8.93	.867
7 SWLS	.49**	.53**	.51**	.44**	.42**	–.29**	1	29.90	5.72	.831

Note: M (average), PA (positive affect), NA (negative affect), and SWLS (satisfaction with life); **p = .001

In order to test the model, the next step was its specification, in which the relationships among the variables were established. For the identification phase, the parameters were estimated, and due to the non-normal distribution of the multivariate data, given the level of significance of *p* = .05, it could be concluded that the variables, as a whole, presented a significantly different kurtosis from that of a multivariate normal distribution (Mardia = 46.36, c.r. 43.80). The maximum likelihood method was used, since it is a method robust to deviations from normality (Abad et al., 2011). The model was configured with the interrelations between the latent variables and the indicators of psychological adjustment, namely positive affect, negative affect, and satisfaction with life. In the first model, the psychological adjustment indicators revealed that the model was generally acceptable; however, measures indicative of the quality of model fit were analyzed and modification indices were considered, which suggested including a covariance term between errors 1 and 2, which corresponded to PA and NA. Thus, we proceeded to perform this adjustment, which would improve the fit of the model (Sörbom, 1989) without losing the theoretical value.

The final model showed the following absolute fit indices: CMIN = 76.26, DF = 8, CMIN/DF = 9.53. However, other indices were also examined, since the model fit is usually affected by the sample size (Abad et al., 2011). The other absolute fit indices that were taken into account included GFIwith a value of .955, which was greater than .90, indicating that the model had a good fit and was recommended, and AGFI with a value of .843, which was lower than the recommended value of .90 or higher. The mean square error indicated a bad fit, as RMSEA = .138, which was higher than the recommended value of .08. The RMR index was .0519, which was adequate (Arbuckle, 2012). The Comparative Adjustment Index (CFI= .937) and the Normative Adjustment Index (NFI= .931) were both higher than .90 (Bentler, 1990). The parsimony adjustment indices were within the magnitude considered acceptable (PGFI= .27 and IFI= .938). With the absolute fit indices and parsimony adjustment indices indicating a relatively adequate fit, the re-specified model was considered relevant (Hu & Bentler, 1998) (*Table 4*).

**Table 4:** Goodness-of-fit indices of the hypothesized model

	CMIN	DF	CMIN/DF	GFI	AGFI	RMSEA	RMR	CFI	NFI	PGFI	IFI
1	11.60	9	12.28	.939	.809	.159	.0530	.906	.900	.302	.907
2	76.26	8	9.53	.955	.843	.138	.0519	.937	.931	.270	.938

**Figure 1:**
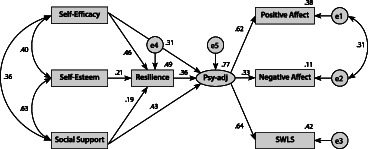
Structural equation model of the role of self-esteem, self-efficacy, and family social support in resilience and psychological adjustment

*Figure 1* shows the final model with the standardized solution values, and all the interrelations were found to be significant. The model indicates that self-esteem, self-efficacy, and family social support are associated with resilience and affect the psychological adjustment indicators; in addition, family social support has a significant direct effect on the dependent variable.

## Discussion

This study had four objectives; the first was to verify differences in the variables under study according to the levels of resilience shown by the children. The results indicate that participants who show very high levels of resilience have higher scores in self-esteem, self-efficacy, and family social support, as well as better psychosocial adjustment. This is consistent with the findings reported in previous studies by Gutiérrez and Romero (2014), Ramos-Díaz (2015), and Gómez-Baya et al. (2019), as these are the characteristics of resilient people (González Arratia et al., 2022).

Regarding the second study objective, the findings indicate that there are no significant differences between boys and girls as hypothesized. Harter (1982) has also shown that levels of self-esteem are similar for boys and girls, particularly those between the ages of 10 and 11. The classic studies by Coopersmith (1967) indicated that differences in self-esteem according to sex mainly emerge from adolescence onwards. Similarly, according to the study by Alcaide-Risoto et al. (2017) with Spanish primary school children, there are no major differences between boys and girls in terms of self-esteem, which changes in adolescence. Another finding of the present study is that no significant differences were found between boys and girls with respect to self-efficacy; like self-esteem, adolescence is a transitional stage in which a decrease in self-efficacy can be expected, and a difference in self-efficacy between males and females may be seen (Carrasco Ortíz & Del Barrio Gándara, 2002; Maldonado et al., 2020). Nevertheless, there are still no conclusive data regarding the differences in levels of self-efficacy between males and females (Navarro et al., 2019). Although research on self-efficacy in children and adolescents has gained relevance, only global self-efficacy was investigated in the present study, so it would be pertinent to analyze self-efficacy in different domains (academic, social, and self-regulatory) to investigate whether there is a decrease and a change in self-efficacy from adolescence onwards. Regarding family social support, no significant differences were found, which is in line with the study by Rodríguez Espínola (2010). Therefore, the results of this study suggest that variations in these variables according to sex require further analysis. In terms of resilience, there are also no differences between the sexes, indicating a need for further research on any differences in resilience between boys and girls. Regarding differences in psychological adjustment between males and females, several investigations have been conducted but have not reached an agreement on the matter. Authors such as Fernández-Daza and Fernández Parra (2017) found that boys show more problems related to psychological adjustment than girls. On the other hand, a study by Rodrigues et al. (2019) indicated the opposite. However, in the present research, there are no differences, and the reason for the presence or the lack of differences is an important future research direction.

The correlation analyses showed significant positive and negative relationships between the variables, with magnitude ranging from low to moderate (Rivera & García, 2012), which coincides with the findings of the study by González Arratia et al. (2020). It is necessary to point out that the magnitude of the relationship between social support and satisfaction with life obtained in this study is slightly higher than what has been reported by González Arratia et al. (2020), which suggests that although these variables are associated with each other, variations in the magnitude of the relationships may be due to factors such as the way the questionnaires were administered in the present study; this would have to be verified in future research.

With regard to the proposed model, which was verified, the data of the present study reveal that self-esteem, self-efficacy, and family social support predict resilience; in particular, self-efficacy is the variable that best explains resilience (Olivari & Urra, 2007), while perceived family social support has a significant direct effect on psychosocial adjustment, suggesting a determining role of social support in psychological adjustment (Ramos-Díaz, 2015). Given the significant relationships among the variables under study, it is evident that resilience plays an important role in psychological adjustment, which implies that children with higher self-esteem, self-efficacy, and family social support are more likely to experience greater satisfaction with life, greater positive affect, and lower negative affect; these results contribute to the evidence that they are indicators of psychological adjustment in childhood (Fuentes et al., 2011).

## Conclusion

In general, the proposed model was found to be relatively acceptable, and the estimation of variances and covariances as accurately as possible in this study facilitated the explanation and prediction of the proposed theoretical model. The results of this study account for the complex interactions between the variables under study, but it is necessary to obtain further theoretical and empirical evidence, as well as to test other models, where personal and contextual variables are included to better understand the variables influencing resilience and psychological adjustment and identify key elements for the development of intervention from the perspective of positive psychology.

## Limitations

A limitation of this study is the design of the study itself; since it was a cross-sectional study, the data were obtained from a specific time point, and because resilience is a dynamic construct, it is necessary to follow up with the sample in order to understand its complex interactions with other variables in explaining psychological adjustment in different moments of crisis, especially with the emergence of adolescence. Another limitation is that the sample was a non-random sample; therefore, it is necessary to analyze the study variables in an expanded sample, which will allow us to continue testing the proposed model and other plausible models for their explanation of psychological adjustment.
